# A Novel Approach for Solving the BMI Problem in Barrier Certificates Generation

**DOI:** 10.1007/978-3-030-53288-8_29

**Published:** 2020-06-13

**Authors:** Xin Chen, Chao Peng, Wang Lin, Zhengfeng Yang, Yifang Zhang, Xuandong Li

**Affiliations:** 8 Microsoft Research Lab, Redmond, WA USA; 9 University of Southern California, Los Angeles, CA USA; 10 State Key Laboratory for Novel Software Technology, Nanjing University, Nanjing, China; 11 Shanghai Key Lab of Trustworthy Computing, East China Normal University, Shanghai, China; 12 School of Information Science and Technology, Zhejiang Sci-Tech University, Hangzhou, China

**Keywords:** Formal verification, Hybrid systems, Barrier certificates, Bilinear matrix inequalities

## Abstract

Barrier certificates generation is widely used in verifying safety properties of hybrid systems because of the relatively low computational complexity it costs. Under sum of squares (SOS) relaxation, the problem of barrier certificate generation is equivalent to that of solving a bilinear matrix inequality (BMI) with a particular type. The paper reveals the special feature of the problem, and adopts it to build a novel computational method. The proposed method introduces a sequential iterative scheme that is able to find analytical solutions, rather than the nonlinear solving procedure to produce numerical solutions used by general BMI solvers and thus is more efficient than them. In addition, different from popular LMI solving based methods, it does not make the verification conditions more conservative, and thus reduces the risk of missing feasible solutions. Benefitting from these two appealing features, it can produce barrier certificates not amenable to existing methods, which is supported by a complexity analysis as well as the experiment on some benchmarks.

## Introduction

Cyber-physical systems (CPS) consists of tightly coupled physical components such as electrical, mechanical, hydraulic, and biological components and software systems. They are deeply involved in many safety-critical systems, for example, high confidence medical devices, traffic control and safety systems, advanced automotive systems and critical infrastructure control systems. Safety verification helps to ensure them not to behave dangerously.

Hybrid systems are popular models used in the verification of Cyber-physical systems, for its ability to describe interacting discrete transitions and continuous dynamics [18]. Safety verification contributes to checking safety properties by determining whether a system can evolve to some states violating desired safety properties when it starts at some initial conditions. A successful verification of a hybrid system can raise our confidence in its corresponding Cyber-physical system.

For Cyber-physical systems with real time constraints, fast verification is a vital requirement. For example, a online verification module in a monitoring system should return the result before the deadline is reached. The paper aims at fast verification of hybrid systems to satisfy the requirement of fast verification of Cyber-physical systems.

Intuitively, safety verification of hybrid systems can be performed by computing the reachable set. Reachable set computation based approaches explicitly computes either exact or approximate reachable sets corresponding to the dynamics in the model, and then compares them with unsafe regions. It has been successfully adopted in verifying behaviors of a system within a finite horizon. However, due to their intrinsic computational difficulty, approaches of this kind can hardly scale up to complex non-linear systems.

Many research efforts have been devoted to barrier certificate generation. A barrier certificate is a function, of which the zero level set separates the unsafe region from all reachable states of a system. It requires all system trajectories starting from some initial conditions fall into one side of the barrier certificate while the unsafe region resides on the other. As the existence of a barrier certificate implies that the unsafe region is not reachable, the safety verification problem can be transformed into the problem of barrier certificate generation. Compared with reachable set computation [31], barrier certificate generation requires much less computation, since the unsafe region leads to seeking a barrier certificate. Especially, it behaves very well when a safety property concerns infinite time horizon [21, 34].

Barrier certificate generation is a computation intensive task. A set of verification conditions corresponding to a specific type of barrier certificates is given at first. Then they are encoded into some constraints on state variables and unknown coefficients of barrier certificates of a specific type. Finally, those unknown coefficients are determined by solving the constraints [27]. Thus, how to encode verification conditions and solve them in an effective way is a critical and challenging problem in barrier certificate based verification.

Acting as the barrier between reachable states and the unsafe region, a barrier certificate should always evaluate to be nonnegative or negative accordingly in spite of what type it is. To achieve this, the most popular computational method utilizes the theory of Putinar’s Positivstellensatz to derive a *sum of squares* (SOS) program of the barrier certificate, which results in a *bilinear matrix inequality * (BMI) solving problem belonging to the class of NP-hard problems [20, 21]. An effective and efficient BMI solver is a prerequisite for success in exploiting SOS relaxation based methods.

The general BMI problem can be solved by the commercial BMI solver PENBMI [14] at the cost of a very high computational complexity, where the (exterior) penalty and (interior) barrier method incorporates with the augmented Lagrangian method. To make it more tractable, the convex SOS relaxation based methods become popular. They transform the BMI problem (non-convex) to a *linear matrix inequality * (LMI) problem (convex) by fixing some multipliers and then solve it quickly via convex optimization such as semidefinite programming (SDP). Unfortunately, the removal of non-convexity may yield too conservative verification conditions so that the solution to the original BMI problem is invisible to the derived LMI problem.

The paper focuses on quickly solving the BMI problem derived from SOS relaxation by directly attacking the problem without relaxing it to a LMI one. Taking advantage of the special feature of the problem, that is all bilinear terms are cross ones between different parameter vectors, a sequential iterative scheme is proposed. It treats the non-convex BMI problem directly so as to avoid the loss of precision accompanied with non-convexity removing. Meanwhile, it provides much lower computational complexity than the PENBMI solver. Hence, the proposed method spends much less time in computation and has the potential to find solutions beyond the reach of existing methods.

To be specific, a feasible solution to the BMI problem can be found by a dual augmented Lagrangian iterative framework. At each iteration, the minimization over the four sets of primal variables is divided into four sequential minimization problems with respect to one set of primal variables by fixing the other three sets. On the theoretical side, we show that our method returns the feasible solution in cubic time, while the PENBMI solver in quartic time. We have developed a prototyping tool implementing the proposed method and compared it with the PENBMI solver and the LMI solver: SOSTOOLS [22] over a set of benchmarks gathered from the literature. The experiment shows that our tool is more effective than them and provides a much lower computational complexity than the PENBMI solver.

The paper is organized as follows. Section 2 describes the connection between safety verification and barrier certificate generation. Section 3 addresses how to transform the problem of barrier certificate generation into a BMI solving problem. In Sect. 4, a sequential iterative scheme is presented followed by a complexity analysis. Section 5 contains detailed examples illustrating the use of our method as well as the experiment on benchmarks. We compare with related works in Sect. 6 before concluding in Sect. 7.

## Preliminaries

**Notations.** Let ${\mathbb R}$ be the field of real number. ${\mathbb R}[{\mathbf{x}}]$ denotes the polynomial ring with coefficients in ${\mathbb R}$ over variables ${\mathbf{x}}=[x_1,x_2,\cdots ,x_n]^T$. Let $\varSigma [{\mathbf{x}}]\subset {\mathbb R}[{\mathbf{x}}]$ be the space of SOS polynomials. $S^{n}$ denotes the set of $n\times n$ symmetric matrices, and the notation $B \succeq 0$ means that the matrix $B\in S^{n}$ is positive semidefinite. $\langle A,B \rangle$ denotes the inner product between *A* and *B*.

A continuous dynamical system is modeled by a finite number of first-order ordinary differential equations

1 $$\begin{aligned} \dot{{\mathbf{x}}}={\mathbf{f}}({\mathbf{x}}), \end{aligned}$$

where $\dot{{\mathbf{x}}}$ denotes the derivative of ${\mathbf{x}}$ with respect to the time variable *t*, and ${\mathbf{f}}({\mathbf{x}})$ is called vector field ${\mathbf{f}}({\mathbf{x}})=[f_1({\mathbf{x}}),\cdots , f_n({\mathbf{x}})]^T$ defined on an open set $\varPsi \subseteq {\mathbb R}^{n}$. We assume that ${\mathbf{f}}$ satisfies the local Lipschitz condition, which ensures that given ${\mathbf{x}}={\mathbf{x}}_0$, there exists a time $T>0$ and a unique function $\tau : [0,T)\mapsto {\mathbb R}^{n}$ such that $\tau (0)={\mathbf{x}}_0$. And ${\mathbf{x}}(t)$ is called a solution of (1) that starts at a certain initial state ${\mathbf{x}}_0$, that is, ${\mathbf{x}}(0)={\mathbf{x}}_0$. Namely, ${\mathbf{x}}(t)$ is also called a trajectory of (1) from ${\mathbf{x}}_0$.

### Definition 1 (Continuous System)

A continuous system over ${\mathbf{x}}$ consists of a tuple ${\mathbf{S}}:\langle \varTheta , {\mathbf{f}},\varPsi \rangle$, wherein $\varTheta \subseteq {\mathbb R}^{n}$ is a set of initial states, ${\mathbf{f}}$ is a vector field over the domain $\varPsi \subseteq {\mathbb R}^{n}$.

A hybrid system is a system which exhibits mixed discrete-continuous behaviors. A popular model for representing hybrid systems is hybrid automata [1], which combine finite state automata modeling the discrete dynamics, and differential equations modeling the continuous dynamics.

### Definition 2 (Hybrid Automata)

A *hybrid automaton* is a tuple ${\mathbf{H}}: \langle L$, *X*, *F*, $\varPsi , E, \varXi , \varDelta , \varTheta , \ell _0\rangle$, where

- *L*, a finite set of locations (or models);
- $X\subseteq {\mathbb R}^n$ is the continuous state space. The hybrid state space of the system is defined by $\mathcal {X}=L\times X$ and a state is defined by $(\ell ,{\mathbf{x}})\in \mathcal {X}$;
- $F: L\rightarrow ({\mathbb R}^n\rightarrow {\mathbb R}^n)$, assigns to each location $\ell \in L$ a locally Lipschitz continuous vector field ${\mathbf{f}}_{\ell }$;
- $\varPsi$ assigns to each location $\ell \in L$ a *location condition (location invariant)* $\varPsi (\ell )\subseteq {\mathbb R}^n$;
- $E\subseteq L\times L$ is a finite set of discrete transitions;
- $\varXi$ assigns to each transition $e\in E$ a switching guard $\varXi _e\subseteq {\mathbb R}^n$;
- $\varDelta$ assigns to each transition $e\in E$ a reset function $\varDelta _e:{\mathbb R}^n\rightarrow {\mathbb R}^n$;
- $\varTheta \subseteq {\mathbb R}^n$, an initial continuous state set;
- $\ell _0\in L$, the initial location. The initial state space of the system is defined by $\ell _0\times \varTheta$.

Trajectories of hybrid systems combine continuous flows and discrete transitions. Concretely, a trajectory of ${\mathbf{H}}$ is an infinite sequence of states $\sigma =\{s_0,s_1,s_2,\cdots \}$ such that

- **[Initiation]** $$s_0=(\ell _0, {\mathbf{x}}_0)$$, with $${\mathbf{x}}_0\in \varTheta $$;
  Furthermore, for each pair of consecutive state $(s_i,s_{i+1})\in \sigma$ with $s_i=(\ell _i,{\mathbf{x}}_i)$ and $s_{i+1}=(\ell _{i+1},{\mathbf{x}}_{i+1})$ satisfies the following one of the two *consecution* conditions:
- **[Discrete Consecution]** $e=(\ell _i,\ell _{i+1})\in E$, ${\mathbf{x}}_i\in \varXi _e$ and $x_{i+1}=\varDelta _e({\mathbf{x}}_i)$;
- **[Continuous Consecution]** $\ell _i=\ell _{i+1}$, and there exists a time interval $\delta >0$ such that the solution ${\mathbf{x}}({\mathbf{x}}_i;t)$ to $\dot{{\mathbf{x}}}={\mathbf{f}}_{\ell _i}$ evolves from ${\mathbf{x}}_i$ to ${\mathbf{x}}_{i+1}$, while satisfying the location invariant $\varPsi (\ell _i)$. Formally, ${\mathbf{x}}({\mathbf{x}}_i,\delta )={\mathbf{x}}_{i+1}$ and $\forall t \in [0,\delta ], {{\mathbf{x}}({\mathbf{x}}_i,t)\in \varPsi (\ell _i)}$.

If $\varSigma$ is the set of all possible trajectories of ${\mathbf{H}}$, the reachable set is defined by $R=\{s|\exists \varsigma \in \varSigma : s\in \varsigma \}$, i.e., *R* contains all states that are elements of at least one trajectory $\varsigma$.

In this paper, we focus on semi-algebraic hybrid systems, that is, the corresponding vector fields are polynomials and the sets $\varTheta , \varPsi (\ell ), \varXi _{e}, \varDelta _{e}$ in ${\mathbf{H}}$ are semi-algebraic, represented by polynomial equations and inequalities. The semi-algebraic sets $\varTheta$, $\varPsi (\ell )$, $\varXi _e$, and $\varDelta _e$ in Definition 2 are represented as follows:

$$\begin{aligned} \left\{ \begin{array}{rlrl} \varTheta :&{}=\{{\mathbf{x}}\in {\mathbb R}^{n}\,|\,\theta ({\mathbf{x}})\ge 0\},\\ \varPsi (\ell ):&{}=\{{\mathbf{x}}\in {\mathbb R}^{n}\,|\,\psi _{\ell }({\mathbf{x}})\ge 0\},\\ \varXi _e:&{} =\{{\mathbf{x}}\in {\mathbb R}^{n}\,|\,\rho _{e}({\mathbf{x}})\ge 0\}, \\ \varDelta _e:&{} =\{{\mathbf{x}}'\in {\mathbb R}^{n}\,|\, \delta _{e}({\mathbf{x}}')\ge 0\}, \end{array} \right. \end{aligned}$$

where $\ell \in L$, $e\in E$, $\theta ({\mathbf{x}})$, $\psi _{\ell }({\mathbf{x}})$, $\rho _{e}({\mathbf{x}})$, and $\delta _{e}({\mathbf{x}}')$ are vectors of polynomials, and the inequalities are satisfied entry-wise. Suppose that $X_u$ assigns to each location $\ell \in L$ an unsafe region $X_u(\ell )$, defined by

$$\begin{aligned} X_u(\ell ): =\{{\mathbf{x}}\in {\mathbb R}^{n}\,|\,\zeta _{\ell }({\mathbf{x}})\ge 0\}, \end{aligned}$$

where $\zeta _{\ell }$ is a vector of polynomials. The safety specification is described over the trace of state $(\ell ,{\mathbf{x}})$ w.r.t. unsafe regions $X_u(\ell )$.

### Definition 3 (Safety)

Given a hybrid system ${\mathbf{H}}: \langle L$, *X*, *F*, $\varPsi , E, \varXi , \varDelta , \varTheta , \ell _0\rangle$ and unsafe regions $X_u(\ell )$, the safety property holds if there exist no trajectories of ${\mathbf{H}}$ starting from the initial set $\ell _0 \times \varTheta$, can evolve to any state specified by $X_u(\ell )$, i.e., $\forall \ell \in L\,\forall \sigma \in \varSigma .\, s\in \sigma \models s\notin X_u(\ell )$.

For safety verification of hybrid systems, the notion of barrier certificates [21] plays an important role. A barrier certificate maps all the states in the reachable set *R* to non-negative reals and all the states in the unsafe region to negative reals, thus can be employed to prove safety of hybrid systems. However, the exact reachable set *R* is usually intractable for most hybrid systems. In [21], a sufficient inductive condition for barrier certificates is defined as follows.

### Definition 4 (Barrier Certificate)

A barrier certificate of hybrid system ${\mathbf{H}}$ for safety w.r.t. unsafe regions $X_u(\ell )$ is a set of real functions $\{B_{\ell }({\mathbf{x}})\}$ such that, for all $\ell \in L$ and $e=(\ell ,\ell ')\in E$, the following conditions hold:

2 $$\begin{aligned} \left\{ \begin{array}{l@{}l} &{}\forall {\mathbf{x}}\in \varTheta :\,B_{\ell _0}({\mathbf{x}}) \ge 0,\\ &{}\forall {\mathbf{x}}\in \varPsi (\ell ):\,B_{\ell }({\mathbf{x}})=0 \models \big \langle \frac{\partial B_\ell }{\partial {\mathbf{x}}}({\mathbf{x}}),{\mathbf{f}}_\ell ({\mathbf{x}})\big \rangle >0,\\ &{}\forall {\mathbf{x}}\in \varXi _e,\forall {\mathbf{x}}'\in \varDelta _e({\mathbf{x}}):\,B_{\ell }({\mathbf{x}})\ge 0 \models B_{\ell '}({\mathbf{x}}')\ge 0,\\ &{}\forall {\mathbf{x}}\in X_u(\ell ):\,B_{\ell }({\mathbf{x}}) < 0. \end{array}\right. \end{aligned}$$

Note that $\big \langle \frac{\partial B_\ell }{\partial {\mathbf{x}}}({\mathbf{x}}),{\mathbf{f}}_\ell ({\mathbf{x}})\big \rangle$ is the Lie derivative of $B_{\ell }({\mathbf{x}})$ with respect to the vector field ${\mathbf{f}}_{\ell }({\mathbf{x}})$.

## Transfer to BMI

The problem of generating barrier certificates in Definition 4 is an infinite-dimensional problem. In order to make it amenable to polynomial optimization, the barrier certificate $\{B_\ell ({\mathbf{x}})\}$ should be restricted to a set of polynomials with a priori degree bound. Putinar’s Positivstellensatz provides a powerful representation for polynomial positivity on semi-algebraic sets, which helps to transform the problem of barrier certificate generation into solving a semidefinite programming via SOS relaxation.

Arising from the second and third conditions of Definition 4, where the parameters of $\{B_\ell ({\mathbf{x}})\}$ appear on the antecedent sides, the associated SOS representations using Putinar’s Positvstellensatz form non-convex BMI constraints, yielded from the polynomial products between the barrier certificate and its polynomial multipliers.

In what follows, the procedure for transforming barrier certificate generation into BMI solving is recapped in detail. Firstly, SOS relaxation is applied to encode the entailment checking in condition (2) as an SOS program. In fact, all the conditions of Definition 4 can be expressed as a unified type, say, a polynomial is nonnegative (positive) on a semi-algebraic set, which can be characterized by Putinar’s Positivstellensatz.

Let ${\mathbb K}$ be a basic semi-algebraic set defined by:

3 $$\begin{aligned} {\mathbb K}=\{{\mathbf{x}}\in {\mathbb R}^{n}\, | \, g_{1}({\mathbf{x}})\ge 0,\ldots , g_{s}({\mathbf{x}})\ge 0\}, \end{aligned}$$

where $g_{j}\in {\mathbb R}[{\mathbf{x}}], 1\le j\le s$. Given the finite family ${\mathbf{g}}=\{g_1({\mathbf{x}}),\ldots ,g_{s}({\mathbf{x}})\}$,the polynomial set defined by

$$M({\mathbf{g}})= :=\{\sigma _{0}+\sum _{i=1}^{s}\sigma _{i}g_{i}\,|\,\,\sigma _{i}\in \varSigma [{\mathbf{x}}],\, 0\le i \le s\}$$

is called the quadratic module generated by ${\mathbf{g}}$.

### Theorem 1

[Putinar’s Positivstellensatz] Let ${\mathbb K}\subset {\mathbb R}[{\mathbf{x}}]$ be as in (3). Assume that the quadratic module $M({\mathbf{g}})$ is archimedean, namely, there exists $u({\mathbf{x}}) \in M({\mathbf{g}})$ such that the set $\{{\mathbf{x}}\in {\mathbb R}^{n} | u({\mathbf{x}}) \ge 0\}$ is compact. If $f({\mathbf{x}})$ is strictly positive on ${\mathbb K}$, then $f({\mathbf{x}})$ can be represented as

4 $$\begin{aligned} f({\mathbf{x}})=\sigma _{0}({\mathbf{x}})+\sum _{i=1}^{s}\sigma _{i}({\mathbf{x}})g_{i}({\mathbf{x}}), \end{aligned}$$

where $$\sigma _{i} \in \varSigma [{\mathbf{x}}], 0\le i\le s$$.

Following Theorem 1, the existence of the representation (4) provides a sufficient and necessary condition of polynomial positivity on a semi-algebraic set ${\mathbb K}$ [23]. Although the number of auxiliary polynomials in the representation (4) is only one more than the number of polynomials that define ${\mathbb K}$, the degree bound for $\sigma _{i}({\mathbf{x}})$ is exponential with *n* and $\deg ({\mathbf{f}})$. From a computational point of view, the method for finding the above representation has some degree of conservativeness, say, by fixing a priori much smaller degree bound *D* for $\sigma _{i}({\mathbf{x}})$. Thus, a sufficient condition for the nonnegativity of the given polynomial $f({\mathbf{x}})$ on the semi-algebraic set ${\mathbb K}$ is provided as

5 $$\begin{aligned} f({\mathbf{x}})=\sigma _{0}({\mathbf{x}})+\sum _{i=1}^{s}\sigma _{i}({\mathbf{x}})g_{i}, \end{aligned}$$

with $\deg (\sigma _{i})\le D, \, \sigma _{i} \in \varSigma [{\mathbf{x}}], 1\le i \le s$. The representation (5) ensures that a polynomial is nonnegative on a given semi-algebraic set. At this point, all conditions in Definition 4 can be derived as a unified type, i.e., polynomial nonnegativity on a semi-algebraic set. The representation (5) is used to characterize the conditions of barrier certificate generation, for they are more tractable.

### Theorem 2

Let the semi-algebraic hybrid system ${\mathbf{H}}$ and the unsafe regions $X_u(\ell )$ be defined as the above. Let *D* be a positive integer. Suppose there exist polynomials $\{B_{\ell }({\mathbf{x}})\}$ and $\{\nu _{\ell }({\mathbf{x}})\}$ with $\deg (\nu _{\ell })\le D$, positive numbers $\epsilon _{\ell ,1}$ and $\epsilon _{\ell ,2}$, and vectors of sums of squares $\sigma ({\mathbf{x}})$, $\lambda _{e,i}({\mathbf{x}})$, $\gamma _{e}({\mathbf{x}})$, $\eta _{e}({\mathbf{x}})$, $\phi _{\ell }({\mathbf{x}})$, $\mu _{\ell }({\mathbf{x}})$ with the degree bound *D*, such that the following expressions:

6 $$\begin{aligned} \begin{array}{rl} &{} \quad \,\,\, B_{\ell _0}({\mathbf{x}})-\sigma ({\mathbf{x}})\theta ({\mathbf{x}}) \\ &{}\quad \, \,\,B_{\ell '}({\mathbf{x}}')-\lambda _{e}({\mathbf{x}})\rho _{e}({\mathbf{x}})-\gamma _{e}({\mathbf{x}}')\delta _{e}({\mathbf{x}}') -\eta _{e}({\mathbf{x}})B_\ell ({\mathbf{x}})\\ &{}\quad \,\,\,\,\, \big \langle \frac{\partial B_\ell }{\partial {\mathbf{x}}}({\mathbf{x}}),{\mathbf{f}}_\ell ({\mathbf{x}})\big \rangle -\phi _{\ell }({\mathbf{x}})\psi _{\ell }({\mathbf{x}}) -\nu _{\ell }({\mathbf{x}})B_\ell ({\mathbf{x}})-\epsilon _{\ell ,1}\\ &{}\quad \, -B_\ell ({\mathbf{x}})-\mu _{\ell }({\mathbf{x}}) \zeta _{\ell }({\mathbf{x}})-\epsilon _{\ell ,2} \end{array} \end{aligned}$$

are SOSes for each $\ell \in L$ and $e\in E$. Then $\{B_{\ell }({\mathbf{x}})\}$ satisfies the conditions in Definition 4, and therefore guarantees the safety of ${\mathbf{H}}$.

Remark that a polynomial $f({\mathbf{x}})$ with $\deg (f)=2d$ is a sum of squares if and only if there exists a real symmetric and positive semidefinite matrix *Q*, called as the Gram matrix, such that $f({\mathbf{x}})={\mathbf{v}}_{d}({\mathbf{x}})^{T}Q {\mathbf{v}}_{d}({\mathbf{x}})$, where ${\mathbf{v}}_{d}({\mathbf{x}})$ is the vector consisting of all the monomials of degree less than or equal to *d*. In view of the conditions (6) in Theorem 2, the problem of generating the barrier certificates requires introducing the auxiliary (Gram matrices) variables. In fact, the decision variables in the SOS program (6) are the coefficients of all the unknown polynomials in (6), such as $B_\ell ({\mathbf{x}}), \sigma ({\mathbf{x}}),$ $\lambda _{e}({\mathbf{x}})$ and the associated Gram matrices. The polynomial products, i.e., $B_\ell ({\mathbf{x}}) \eta _{e}({\mathbf{x}})$ and $B_\ell ({\mathbf{x}}) \nu _{\ell }({\mathbf{x}})$, derive some quadratic terms of the products of these unknown coefficients, which occur in the second and third constraints of (6). As a consequence, the problem for generating barrier certificates in Theorem 2 derives a non-convex BMI problem. We now show the transformation by a simple example.

### Example 1

Consider the system $\dot{x}=-x$ with location invariant $\varPsi =\{x\in {\mathbb R}: x^2-1\le 0\}$. Suppose the barrier certificate *B*(*x*) with $\deg (B)=1$, we predetermine its template as $B(x)=u_{0}+u_{1} \, x$ with $u_0,u_1 \in {\mathbb R}$ and $u_1\ne 0$. For simplicity, here we consider the second condition in Definition  4, that is, to find *B*(*x*) which satisfies

$$\forall x\in \varPsi : \,B(x)=0\models \frac{\partial B}{\partial x}\cdot (-x)\ge 0.$$

Following the SOS relaxation in (6), we need to find *B*(*x*) such that

7 $$\begin{aligned} \phi _{0}(x):=\frac{\partial B}{\partial x}\cdot (-x)-\phi _1(x)\cdot (1-x^2)-\phi _2(x)\cdot B(x)-\epsilon \end{aligned}$$

and $\phi _{1}(x)$ are SOSes, $\phi _{2}(x)\in {\mathbb R}[x]$, $\epsilon \in {\mathbb R}_{>0}$. We assume that $\phi _1=u_{2}$ and $\phi _{2}=v$, with $u_2 \in {\mathbb R}_{\ge 0}$ and $v \in {\mathbb R}$. Then (7) yields $\phi _{0}(x)=u_2x^2-(u_1v+u_1)x-u_0v-u_2-\epsilon ,$ and its Gram matrix representation $\phi _{0}(x)={\mathbf{v}}_{1}(x)^T\, Q \, {\mathbf{v}}_{1}(x)$, where

$$\begin{aligned} Q=\begin{bmatrix} u_2 &{} -\frac{1}{2}u_1\,v-\frac{1}{2}u_1 \\ -\frac{1}{2}u_1\,v-\frac{1}{2}u_1 &{} -u_0\,v-u_2 -\epsilon \end{bmatrix} \,\, \text {and} \,\, {\mathbf{v}}_{1}(x)=\begin{bmatrix} x \\ 1 \end{bmatrix}. \end{aligned}$$

Since $\phi _0(x)$ and $\phi _1(x)$ must be SOSes, we have $Q\succeq 0$ and $u_{2}\ge 0$, which is equivalent to

$$\begin{aligned} \mathcal {B}(u_0,u_1,u_2,v)=\begin{bmatrix} u_{2} &{} 0 &{} 0 \\ 0 &{} u_2 &{} -\frac{1}{2}u_1\,v-\frac{1}{2}u_1 \\ 0 &{} -\frac{1}{2}u_1\,v-\frac{1}{2}u_1 &{} -u_0\,v-u_2-\epsilon \end{bmatrix} \succeq 0. \end{aligned}$$

Therefore, the requirement that $\phi _0(x)$ and $\phi _1(x)$ are SOSes is translated into the BMI constraint of the form

8 $$\begin{aligned} \mathcal {B}=B_{0,0}+\sum _{i=0}^{2}u_{i}B_{i,0}+vB_{0,1}+\sum _{i=0}^{2}u_{i}v\,B_{i,1} \succeq 0, \end{aligned}$$

where all $B_{i,j}\in S^{3}$ are constant matrices. $\Box$

As illustrated in Example 1, the problem of generating barrier certificates satisfying condition (6) can be transformed into a BMI problem of the form

9 $$\begin{aligned} \begin{array}{l@{}l} &{} \displaystyle \text {Find } {\mathbf{u}}\in {\mathbb R}^p,\,{\mathbf{v}}\in {\mathbb R}^q \\ &{} \text {s.t.}\, \mathcal {B}({\mathbf{u}},{\mathbf{v}})=B_{0,0}+{\displaystyle \sum _{i=1}^{p}}u_iB_{i,0}+{\displaystyle \sum _{j=1}^{q}v_j} B_{0,j}+{\displaystyle \sum _{i=1}^p \sum _{j=1}^q } u_iv_jB_{ij} \succeq 0, \end{array} \end{aligned}$$

where all $B_{i,j}\in S^{t}$ are constant matrices, ${\mathbf{u}}=[u_1, \dots , u_p]^{T}$, ${\mathbf{v}}=[v_1, \dots , v_q]^T$ are parameter coefficients of the unknown polynomials occurring in the original SOS program. Essentially, the BMI problem (9) is NP-hard. To simplify the problem considerably, the canonical approach is to swap ${\mathbf{v}}$, corresponding to the polynomial multipliers $\eta _{e}({\mathbf{x}})$ and $\nu _{\ell }({\mathbf{x}})$, with the fixed vector. This strategy can reduce the BMI constraint into the associated LMI one. Unfortunately, the resulting LMI problem is considerably more conservative than the original BMI one. To be specific, the fixed $\eta _{e}({\mathbf{x}})$ and $\nu _{\ell }({\mathbf{x}})$ may result in too conservative verification conditions that rule out barrier certificates satisfy the non-convex conditions but not the stronger convex conditions.

By investigating (9), we can find a crucial feature of $\mathcal {B}({\mathbf{u}},{\mathbf{v}})$, that is, all cross terms between parameters of ${\mathbf{u}}$ and ${\mathbf{v}}$ are of the form $u_i\,v_j$. The feature motivates us to design a more efficient approach for the specific type of BMI problems.

## A Sequential Iterative Scheme for Solving BMI Problems

The conventional approaches for solving the BMI problem typically employ the augmented Lagrangian iterative framework, wherein each iteration involves two optimization problems for primal and dual variables. Due to the existence of nonlinear terms (quartic terms) in the associated Lagrangian function, the analytical solutions to the first problem do not exist. The iterative-based nonlinear solving procedure is introduced to obtain the numerical solutions which results in a time-consuming computing process.

Observing the BMI problem (9), we can see that all nonlinear terms are the cross terms between ${\mathbf{u}}$ and ${\mathbf{v}}$. As a result, the associated dual augmented Lagrangian function is *quartic* for all variables, but is *quadratic* with respect to each single variable. Having this crucial feature, if we choose one variable as the independent variable and assign the others with fixed values, we may get the problem of minimizing the quadratic function. According to the first-order optimality condition, given a quadratic function $f({\mathbf{x}})$, the sufficient and necessary condition that $\tilde{\mathbf{x}}$ is a minimizer of $f({\mathbf{x}})$ requires that the gradient of $f({\mathbf{x}})$ to be zero at $\tilde{\mathbf{x}}$, i.e., $\nabla f (\tilde{\mathbf{x}})=0$. As a consequence, the analytical solutions to our studied optimization problem can be easily formulated, since the gradient of the associated Lagrangian function is affine.

The analytical optimal solutions can be obtained by calling simple matrix computation, and thus are much more efficient than numerical solutions whose computation relies on complicated nonlinear optimization methods. The computational advantage is further demonstrated by a complexity analysis of our scheme against the existing BMI solving algorithm that combines the (exterior) penalty and (interior) barrier method with the augmented Lagrangian method, presented later in this section.

To utilize the computational advantage of analytical optimal solutions, for the first optimization problem (w.r.t primal variables) involved in each iteration of the augmented Lagrangian iterative framework, rather than using the usual joint minimization for all primal variables, we introduce a sequential minimization scheme, that is, dividing it into four sequential sub-optimization problems over one independent variable while keeping the others fixed. More concretely, the sub-optimization problem with one single primal variable is constructed by replacing the other variables with their optimal solutions obtained from the current iteration (if available) or the last iteration.

This section first introduces an iterative scheme to solve the BMI problem and then illustrates how to derive analytical solutions to the sub-problems in each iteration followed by a complexity analysis against the existing algorithm.

### An Iterative Scheme

We start by presenting a straightforward reformulation of the BMI problem (9) as follows:

10 $$\begin{aligned} \left\{ \begin{array}{ccl} \lambda ^{*}=&{} \min &{} \lambda \\ &{} s.t. &{} Z=\lambda \cdot I+\mathcal {B}({\mathbf{u}},{\mathbf{v}}) \\ &{} &{} Z\succeq 0. \end{array}\right. \end{aligned}$$

Clearly, there exists a feasible solution $({\mathbf{u}},{\mathbf{v}})$ to the BMI problem (9) if and only if the optimal value of problem (10) is non-positive, i.e., $\lambda ^{*} \le 0$. We try to build an iterative scheme for dealing with the optimization problem (10).

The augmented Lagrangian function $\mathcal {L}$ associated with (10) is defined as:

11 $$\begin{aligned}  \mathcal {L}_\mu (\lambda , {\mathbf{u}}, {\mathbf{v}}, Z, U) = \lambda +\langle U, Z-\lambda I - \mathcal {B}({\mathbf{u}},{\mathbf{v}})\rangle +\frac{1}{2\mu }\Vert Z-\lambda I - \mathcal {B}({\mathbf{u}},{\mathbf{v}})\Vert _F^2, \end{aligned}$$

where $\mu >0$, $\langle \cdot , \cdot \rangle$ means the inner product operator, and $\Vert \cdot \Vert _F$ denotes the Frobenius norm of a matrix. Let $U \in S^{t}$ be the Lagrangian multiplier associated with the equality constraint, the dual function is defined as

$$\begin{aligned} g(U)= \inf _{(\lambda , {\mathbf{u}}, {\mathbf{v}}, Z)} \mathcal {L}_\mu (\lambda , {\mathbf{u}}, {\mathbf{v}}, Z, U), \end{aligned}$$

and the *Lagrange dual problem* associated with (10) is to maximize this dual function *g*(*U*), i.e., $\displaystyle {\max _{U}\,\,} g(U)$. Clearly, the dual function yields lower bounds on the optimal value $\lambda ^*$ of the problem (10), that is, $g(U)\le \lambda ^*$ for any *U*.

Applying the dual ascent [17] to the augment Lagrangian function yields the iterative scheme, consisting of the following updates

12 $$\begin{aligned} \left. \begin{array}{rl} &{}(\lambda ^{k+1},{\mathbf{u}}^{k+1},{\mathbf{v}}^{k+1},Z^{k+1}):= \, {\displaystyle \mathop {\mathrm {argmin}}\limits _{\lambda , {\mathbf{u}}, {\mathbf{v}}, Z}} \,\, \mathcal {L}_\mu (\lambda , {\mathbf{u}}, {\mathbf{v}}, Z, U^k),\\ &{}\quad \quad \quad \quad \quad \quad \quad \quad \quad \quad \quad \quad \,\, s.t. \quad Z\succeq 0,\\ &{}U^{k+1}:= \,{\displaystyle \mathop {\mathrm {argmax}}\limits _{U}} \,\, {\mathcal {L}_\mu (\lambda ^{k+1}, {\mathbf{u}}^{k+1}, {\mathbf{v}}^{k+1},Z^{k+1}, U)}, \end{array} \right\} \end{aligned}$$

where the first step is the primal variables update, and the second step is the dual variable update.

The first step in (12) consists of quartic terms and is lack of analytical solution. Thus, it requires jointly minimizing $\mathcal {L}_\mu (\lambda , {\mathbf{u}}, {\mathbf{v}}, Z, U^k)$ with respect to $\lambda , {\mathbf{u}}, {\mathbf{v}}$ and *Z*, which can be directly solved by applying the iterative-based nonlinear optimization procedure at the cost of a high computational complexity. Instead of the usual joint minimization solving, we separate the minimization over the primal variables $\lambda , {\mathbf{u}},{\mathbf{v}},Z$ into four steps, that is, $\lambda , {\mathbf{u}}, {\mathbf{v}}$ and *Z* are updated in an alternating scheme, that is, minimizing $\mathcal {L}_{\mu }$ with respect to one primal variable given the others fixed. In detail, the sequential iterative scheme consists of the following new iterations:

13 $$\begin{aligned} \lambda ^{k+1}&:= {\displaystyle \mathop {\mathrm {argmin}}\limits _{\lambda }} \, \mathcal {L}_\mu (\lambda , {\mathbf{u}}^k, {\mathbf{v}}^k, Z^k, U^k), \end{aligned}$$

14 $$\begin{aligned} {\mathbf{u}}^{k+1}&:={\displaystyle \mathop {\mathrm {argmin}}\limits _{{\mathbf{u}}}} \, \mathcal {L}_\mu (\lambda ^{k+1}, {\mathbf{u}}, {\mathbf{v}}^k, Z^k, U^k), \end{aligned}$$

15 $$\begin{aligned} {\mathbf{v}}^{k+1}&:={\displaystyle \mathop {\mathrm {argmin}}\limits _{{\mathbf{v}}}}\, \mathcal {L}_\mu (\lambda ^{k+1}, {\mathbf{u}}^{k+1}, {\mathbf{v}}, Z^k, U^k), \end{aligned}$$

16 $$\begin{aligned} Z^{k+1}&:={\displaystyle \mathop {\mathrm {argmin}}\limits _{Z \succeq 0}} \, \mathcal {L}_\mu (\lambda ^{k+1}, {\mathbf{u}}^{k+1}, {\mathbf{v}}^{k+1}, Z, U^k), \end{aligned}$$

17 $$\begin{aligned} U^{k+1}&:={\displaystyle \mathop {\mathrm {argmax}}\limits _{U}} \, \mathcal {L}_\mu (\lambda ^{k+1}, {\mathbf{u}}^{k+1}, {\mathbf{v}}^{k+1}, Z^{k+1}, U). \end{aligned}$$

The above iterative scheme introduces a sequential minimization that treats the four primal variables one by one. Benefited from the fact that the explicit formulae for the minimizer or maximizer (13–17) are available, the analytical solutions can be directly derived. Furthermore, as the computation of those analytical solutions involves only simple matrix computation, such as eigenvalue decomposition and matrix inverse, it will be very efficient.

### Analytical Solutions for the Sequential Iteration

In this subsection, we focus on how to find analytical solutions to problems (13–17) in terms of the first-order optimality conditions.

#### Theorem 3

The minimizer $\lambda ^{k+1}$ of (13),i.e.,

$$\lambda ^{k+1}:={\displaystyle \mathop {\mathrm {argmin}}\limits _{\lambda }} \,\, \mathcal {L}_\mu (\lambda , {\mathbf{u}}^k, {\mathbf{v}}^k, Z^k, U^k), $$

has the following analytical formula:

18 $$\begin{aligned} \lambda ^{k+1}:=\frac{1}{t}\sum _{i=1}^t(Z_{i, i}^{k}-\mathcal {B}_{i, i}({\mathbf{u}}^k, {\mathbf{v}}^k))+\frac{\mu }{t}\cdot (\mathrm {Tr}(U^k)-1), \end{aligned}$$

where $\mathrm {Tr}(U^k)$ denotes the trace of $U^k$.

#### Proof

The first-order optimality condition for (13) is

$$\nabla _{\lambda } \mathcal {L}_{\mu }=1-\mathrm{Tr}(U^k)+\frac{t}{\mu }\lambda - \frac{1}{\mu }\sum _{i=1}^t(Z_{i,i}^k-\mathcal {B}_{i,i}({\mathbf{u}}^k,{\mathbf{v}}^k))=0.$$

It follows that the specified $\lambda ^{k+1}$ in (18) is the optimal solution of (13), which concludes the proof. $\Box$

The first-order optimality condition resembling Theorem 3 can also be invoked to produce the corresponding analytical solutions to (14) and (15), respectively.

#### Theorem 4

Let ${\mathbf{v}}^{k}=[v_1^{k},\ldots ,v_{q}^{k}]^{T} \in {\mathbb R}^{q}$, and define $X^{[i]}=B_{i,0}+\sum _{\ell =1}^{q} v_{\ell }^{k} B_{i,\ell }$ for $0\le i \le p.$ Let ${\mathbf{u}}^{k+1}$ be the minimizer of (14). Then

19 $$\begin{aligned} {\mathbf{u}}^{k+1}:=S^{-1}\cdot [r_1,\ldots ,r_p]^{T}, \end{aligned}$$

where $S=[s_{ij}] \in {\mathbb R}^{p\times p}$ with $s_{ij}=\frac{1}{\mu }\langle X^{[i]},X^{[j]}\rangle$, and

$$r_{i}= \langle U^{k}+\frac{1}{\mu }(Z^{k}-\lambda ^{k+1} I-X^{[0]}), X^{[i]}\rangle , 1\le i \le p. $$

#### Proof

The first-order optimality condition for (14) is

$$\begin{aligned} \begin{aligned}&\nabla _{{\mathbf{u}}}\mathcal {L}_\mu (\lambda ^{k+1},{\mathbf{u}},{\mathbf{v}}^{k},Z^{k},U^{k}) =(\nabla _{{\mathbf{u}}_1}\mathcal {L}_\mu , \nabla _{{\mathbf{u}}_2}\mathcal {L}_\mu , \cdots , \nabla _{{\mathbf{u}}_p}\mathcal {L}_\mu )^T=0, \end{aligned} \end{aligned}$$

and the *i*-th gradient function $\nabla _{{\mathbf{u}}_i}\mathcal {L}_\mu (\lambda ^{k+1},{\mathbf{u}},{\mathbf{v}}^k,Z^k,U^k)$, $1\le i \le p$ is

$$\begin{aligned} \begin{aligned}&\langle U^k, -\sum _{\ell =1}^q {\mathbf{v}}_{\ell }^{k}B_{i, \ell }-B_{i,0}\rangle +\frac{1}{\mu }\langle Z^{k}-\lambda ^{k+1} I-\mathcal {B}({\mathbf{u}},{\mathbf{v}}^{k}), -\sum _{\ell =1}^q {\mathbf{v}}_{\ell }^{k} B_{i, \ell }-B_{i, 0}\rangle . \end{aligned} \end{aligned}$$

Then we have

$$\begin{aligned} \begin{aligned}&\nabla _{{\mathbf{u}}_i}\mathcal {L}_\mu (\lambda ^{k+1},{\mathbf{u}},{\mathbf{v}}^{k},Z^{k},U^{K}) = \langle U^{k},-X^{[i]}\rangle +\frac{1}{\mu }\langle Z^{k}-\lambda ^{k+1} I-\mathcal {B}({\mathbf{u}},{\mathbf{v}}^{k}), -X^{[i]}\rangle \end{aligned} \end{aligned}$$

for $$i=1\ldots ,p$$.

Thus, $\nabla _{{\mathbf{u}}}\mathcal {L}_\mu (\lambda ^{k+1},{\mathbf{u}},{\mathbf{v}}^{k},Z^{k},U^{k})=0$ yields (19), which proves the claim. $\Box$

#### Theorem 5

Let ${\mathbf{u}}^{k+1}=[u_1^{k+1},\ldots ,u_{p}^{k+1}]^{T} \in {\mathbb R}^{p}$, and define $Y^{[j]}=B_{0,j}+\sum _{\ell =1}^{p} u_{\ell }^{k+1} B_{\ell ,j}$, for $0\le j \le q$. Let ${\mathbf{v}}^{k+1}$ be the minimizer of (15). Then

20 $$\begin{aligned} {\mathbf{v}}^{k+1}:=T^{-1}\cdot [w_1,\ldots ,w_q]^{T}, \end{aligned}$$

where $T=[t_{ij}] \in {\mathbb R}^{q\times q}$ with $t_{ij}=\frac{1}{\mu }\langle Y^{[i]},Y^{[j]}\rangle$, and

$$w_{i}= \langle U^{k}+\frac{1}{\mu }(Z^{k}-\lambda ^{k+1} I-Y^{[0]}), Y^{[i]}\rangle , \quad 1\le i \le q.$$

#### Proof

Similar to the proof of Theorem 4. $\Box$

The theorems below demonstrate the analytical solutions to the *Z*-minimization and *U*-maximization, respectively.

#### Theorem 6

Let $Z^{k+1}$ be the minimizer of (16), and $U^{k+1}$ be the solution of (17). Denote by $P^{k+1}$ the matrix $P^{k+1}:=\lambda ^{k+1} I + \mathcal {B}({\mathbf{u}}^{k+1}, {\mathbf{v}}^{k+1}) - \mu U^{k}.$ Suppose $P^{k+1}=Q\varSigma Q^{T}$ is a spectral decomposition, namely,

$$P^{k+1}=Q\varSigma Q^{T}=\begin{bmatrix} Q_{\dagger }&Q_{\ddagger }\end{bmatrix} \begin{bmatrix} \varSigma _{+} &{} 0 \\ 0 &{} \varSigma _{-} \end{bmatrix} \begin{bmatrix} Q_{\dagger }^{T} \\ Q_{\ddagger }^{T} \end{bmatrix},$$

where $\varSigma _{+}$ and $Q_{\dagger }$ are the nonnegative eigenvalues and the associated orthogonal eigenvectors, while $\varSigma _{-}$ and $Q_{\ddagger }$ are the negative eigenvalues and the associated orthogonal eigenvectors. Then we have

21 $$\begin{aligned}&Z^{k+1} :=Q_{\dagger }\varSigma _+ Q_{\dagger }^{T},\end{aligned}$$

22 $$\begin{aligned}&U^{k+1} :=-\frac{1}{\mu }Q_{\ddagger }\varSigma _- Q_{\ddagger }^{T}. \end{aligned}$$

#### Proof

The first-order optimality condition for (16) is

23 $$\begin{aligned} \nabla _{Z}\mathcal {L}_\mu (\lambda ^{k+1}, {\mathbf{u}}^{k+1}, {\mathbf{v}}^{k+1}, Z, U^k)=0. \end{aligned}$$

In view of the terms of (23), the problem (16) is translated to

24 $$\begin{aligned} Z^{k+1}=\mathop {\mathrm {argmin}}\limits _{Z\succeq 0} \Vert Z-\lambda ^{k+1} I -\mathcal {B}({\mathbf{u}}^{k+1}, {\mathbf{v}}^{k+1})+\mu U^{k}\Vert _F^2, \end{aligned}$$

which reads as

$$Z^{k+1}=\mathop {\mathrm {argmin}}\limits _{Z\succeq 0} \Vert Z-P^{k+1}\Vert _F^2.$$

According to the spectral decomposition of $P^{k+1}$, the result (21) immediately follows.

From (17), we have

$$\begin{aligned} \begin{aligned} U^{k+1}&=U^k+\frac{1}{\mu }(Z^{k+1}-\lambda ^{k+1} I - \mathcal {B}({\mathbf{u}}^{k+1}, {\mathbf{v}}^{k+1}))\\&=\frac{1}{\mu }(Z^{k+1}-P^{k+1}), \end{aligned} \end{aligned}$$

which yields the result (22). $\Box$

### Algorithm and Complexity Analysis

From the above observation in Sect. 4.1 and Sect. 4.2, the detailed procedure for the sequential iterative scheme is summarized in Algorithm 1.
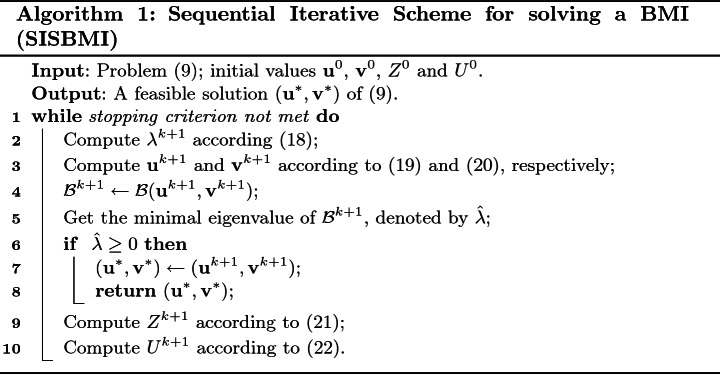


#### Remark 1

At the beginning of Algorithm 1, ${\mathbf{u}}^0\in {\mathbb R}^p$, ${\mathbf{v}}^0\in {\mathbb R}^q$ are selected randomly, $Z^0=M_0^{\top }\cdot M_0$ where $M_0 \in {\mathbb R}^{t}$ is chosen randomly, and heuristically $U^0 = \delta \cdot I_t$ with $\delta >0$.

#### Remark 2

There are several options for the stopping criterion of the loop in Algorithm 1. That is, Algorithm 1 will stop and return the current result when one of the following cases occurs:

- $$|\lambda ^{k+1} -\lambda ^{k} | \le \epsilon $$,
- $$\Vert Z^{k+1} -Z^k \Vert \le \epsilon $$,

where $\epsilon$ is a given tolerance. A reasonable value for the stopping criterion might be $\epsilon =10^{-6}$.

**Complexity Analysis**

We analyze the complexity of Algorithm 1 and further compare it with the algorithm in PENBMI solver [14], which combines the (exterior) penalty and (interior) barrier method with the augmented Lagrangian method. The BMI problem we study corresponds to a nonconvex optimization problem with quartic terms. For the BMI problems of the special form, neither of the two algorithms can guarantee to converge. A complete complexity analysis is not available as the number of iterations is not predictable. Therefore, the computational complexity of one iteration becomes a safe baseline for performance evaluation. In this paper, we follow the same complexity analysis as that in [14], i.e. analyzing the complexity in one iteration.

Recall that the dimension of the matrix $\mathcal {B}({\mathbf{u}},{\mathbf{v}})$ in (9) is *t*, and the numbers of variables ${\mathbf{u}}$ and ${\mathbf{v}}$ are *p* and *q*, respectively. We see that each iteration in Algorithm 1 can be divided into five steps. Firstly, the step of updating $\lambda$ costs ${ O }(t)$ flops, which is carried out by $3t+3$ adds. In the step of ${\mathbf{u}}-$update, the complexity is clearly dominated by the computation of the inverse of $A_{\mathbf{u}}\in {\mathbb R}^{p\times p}$, which costs $O(p^3)$ flops [5]. Analogously, ${\mathbf{v}}-$update can be done in ${ O }(q^3)$ flops. In the step of $Z-$update, the critical issue is to compute the eigenvalue decomposition of matrix $V^{k+1}\in {\mathbb R}^{t\times t}$, at a cost of about $\frac{4}{3} t^{3}$ flops. So the step of $Z-$update requires ${ O }(t^3)$ flops. Finally, the step of $U-$update requires about ${ O }(t)$ flops by performing $U^{k+1}$.

Now, the complexity for the above steps in each iteration of Algorithm 1 is summarized as follows:

- Calculation of $$\lambda \rightarrow { O }(t)$$;
- Calculation of $${\mathbf{u}}\rightarrow { O }(p^3)$$;
- Calculation of $${\mathbf{v}}\rightarrow { O }(q^3)$$;
- Calculation of $$Z\rightarrow { O }(t^3)$$;
- Calculation of $$U\rightarrow { O }(t)$$.

The total cost of each iteration in Algorithm 1 is then ${ O }(p^3+q^3+t^3)$, while the cost of the algorithm adopted in PENBMI is approximately ${ O }((p+q)t^3+(p+q)^2 t^2+(p+q)^3)$, as shown in [14]. Assume that *p*, *q* and *t* are bounded by $T \in {\mathbb Z}$, i.e., $T=\max \{p,q,t\}$, the complexity of Algorithm 1 is approximately ${ O }(T^3)$, whereas the complexity of PENBMI is approximately ${ O }(T^4)$.

## Experiments

In this section, we first show our method by verifying a nonlinear continuous system and then compare our Sequential Iterative Scheme tool: SISBMI solver with the other two solvers: PENBMI and SOSTOOLS.

### Example 2

Consider the following nonlinear continuous system [28]

$$\begin{aligned} \begin{bmatrix} \dot{x_1}\\ \dot{x_2}\\ \dot{x_3} \end{bmatrix} = \begin{bmatrix} 10(x_2-x_1)\\ x_1(28-x_3)-x_2\\ x_1x_2-\frac{8}{3}x_3 \end{bmatrix} \end{aligned}$$

with the location invariant

$$\begin{aligned} \varPsi =\{{\mathbf{x}}\in \mathbb {R}^3 \,|\, -20\le x_1 ,x_3 \le 20, -20\le x_2 \le 0\}. \end{aligned}$$

It is required to verify that all trajectories of the system starting from the initial set

$$ \varTheta =\{{\mathbf{x}}\in \mathbb {R}^3 \,|\, (x_1+14.5)^2+(x_2+14.5)^2+(x_3-12.5)^2\le 16\} $$

will never enter the unsafe region

$$\begin{aligned} X_u=\{{\mathbf{x}}\in \mathbb {R}^3 \,|\, (x_1+16.5)^2+(x_2+14.5)^2+(x_3-2.5)^2\le 38.44\}. \end{aligned}$$

It suffices to find a barrier certificate $B({\mathbf{x}})$, which satisfies all the conditions in Definition 3. Suppose that the degree of $B({\mathbf{x}})$ is 4, and the degree bound $D=6$. Firstly, we construct a bilinear SOS program (6), which is further transformed into a BMI problem of the form (9) where the dimension of $\mathcal {B}({\mathbf{u}},{\mathbf{v}})$ is 78, and the number of decision variables is 396. By applying our algorithm, we succeed to solve the BMI problem and obtain the following barrier certificate

$$\begin{aligned} B({\mathbf{x}}) = \underbrace{-0.0020x_1^4-0.0013x_3^4-0.0131x_1^2x_3^2-0.0022x_1x_2x_3^2 + \cdots +0.0938x_1+62.5702}_{28\ \ terms}. \end{aligned}$$

**Fig. 1.**
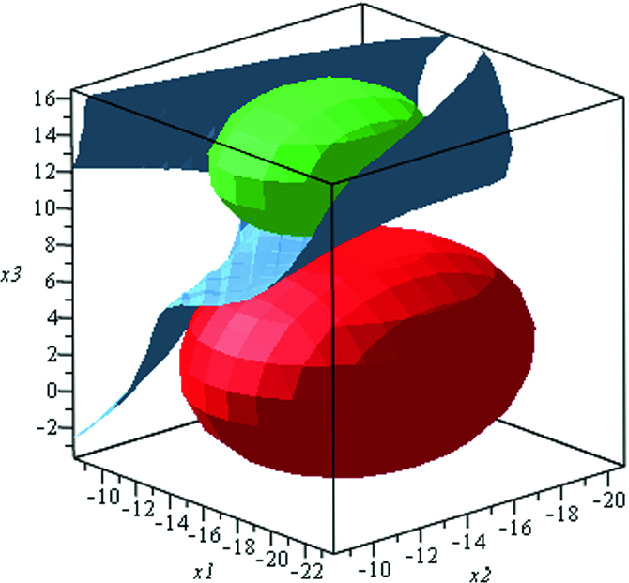
Phase portrait of the system in Example 2. (Color figure online)

As shown in Fig. 1, the zero level set of the barrier certificate $B({\mathbf{x}})$ (the steelblue surface) separates $X_u$ (the red ball) from all trajectories starting from $\varTheta$ (the green ball). Therefore, the safety of the above system is verified.

Alternatively, by applying the PENBMI solver to compute the solution of the problem (9), we cannot find barrier certificates with degree less than 6. $\Box$

### Example 3

**Fig. 2.**
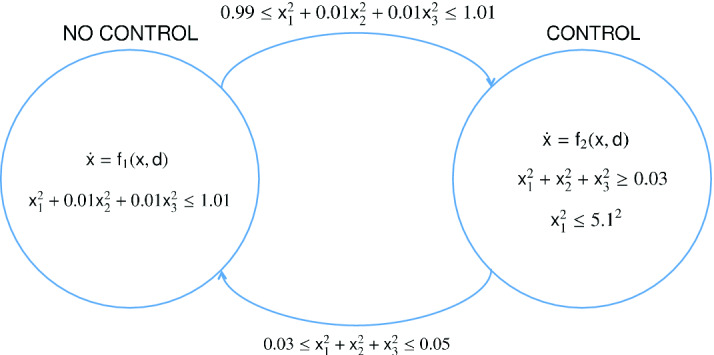
The hybrid automata of the system in Example 3

Consider the following hybrid system [20] depicted in Fig. 2, where

$$\begin{aligned} {\mathbf{f}}_1 = \begin{bmatrix} -x_2\\ -x_1+x_3\\ x_1+(2x_2+3x_3)(1+x_3^2) \end{bmatrix}, \quad {\mathbf{f}}_2 = \begin{bmatrix} -x_2\\ -x_1+x_3\\ -x_1 -2x_2 -3x_3 \end{bmatrix}. \end{aligned}$$

The system starts in location $\ell _1$ with the initial set

$$\begin{aligned} \varTheta =\{{\mathbf{x}}\in \mathbb {R}^3: x_1^2+x_2^2+x_3^2\le 0.01\}. \end{aligned}$$

Our task is to verify that the system will never enter the unsafe set

$$\begin{aligned} X_u(\ell _2)=\{{\mathbf{x}}\in \mathbb {R}^3: 5<x_1<5.1\}. \end{aligned}$$

Applying our SISBMI solver, we obtain the polynomial barrier certificate with degree 4:

$$\begin{aligned} \begin{aligned}&B_{\ell _1}({\mathbf{x}})= \underbrace{0.0551x_1^{4}+0.0392x_2^4+0.0079x_3^4+0.0696x^2x_3^2+\cdots -1.1134x_1+2.701}_{35\ \ terms},\\&B_{\ell _2}({\mathbf{x}})= \underbrace{0.0273{x_1}^4+0.0541x_1^3x_2-1.098x_1x_2^2-0.521x_1x_2x_3+\cdots -2.725x_1+ 8.197}_{35\ \ terms}. \end{aligned} \end{aligned}$$

$$\Box $$

Our SISBMI solver was implemented in Matlab (2018b), and was compared with two solvers PENBMI and SOSTOOLS over a set of benchmarks in the literature on barrier certificates generation. Among these benchmark examples, examples C1–C15 are semi-algebraic continuous systems and examples H1–H7 are semi-algebraic hybrid systems. The performance is reported in Table 1. All the experiments were performed on 2.6 GHz Intel i5 processor under Windows 10 with 8 GB RAM.

**Table 1.** Algorithm performance on benchmarks

ID	*n*	|*L*|	$d_{{\mathbf{f}}}$	BMI								LMI	
				*t*	*N*	SISBMI			PENBMI			SOSTOOLS	
						$d_s$	$I_s$	$T_s$	$d_p$	$I_p$	$T_p$	$d_l$	$T_l$
C1 from [33]	2	1	3	21	33	2	32	0.2189	2	24	0.9198	2	0.1949
C2 from [24]	2	1	1	30	58	4	73	0.5475	—			—	
C3 from [21]	2	1	3	21	39	2	29	0.2761	2	22	1.3353	—	
C4 from [30]	3	1	2	32	72	2	44	0.4126	2	23	1.8237	2	0.3245
C5 from [26]	3	1	3	32	72	2	47	0.4761	2	28	1.5435	2	0.3362
C6 from [3]	3	1	2	78	396	4	83	4.3598	—			—	
C7 from [28]	4	1	3	50	145	2	72	3.9577	2	28	21.0502	2	3.8658
C8 from [9]	3	1	2	32	72	—			2	40	2.4555	—	
C9 from [6]	4	1	2	31	86	—			2	42	4.6909	—	
C10 from [13]	7	1	2	73	394	2	112	10.7156	2	44	108.5615	2	7.2807
C11 from [13]	9	1	2	102	908	2	264	20.6856	2	30	272.4551	2	15.8167
C12 from [8]	12	1	1	70	123	2	108	3.2712	—			—	
H1 from [25]	2	2	2	38	65	2	61	0.4899	2	25	2.1499	2	0.2074
H2 from [36]	2	2	3	42	69	2	77	0.6331	2	24	2.2786	2	0.2265
H3 from [15]	2	2	2	75	138	2	115	3.7394	—			—	
H4 from [2]	2	3	1	42	89	1	70	0.5326	1	21	0.9968	2	0.1856
H5 from [1]	3	3	1	67	64	2	112	1.0864	—			—	
H6 from [7]	4	6	2	840	2736	2	616	48.0548	—			—	
H7 from [20]	3	2	3	170	899	4	219	18.7912	4	32	243.9832	—	

In Table 1, *n* denotes the number of the system variables, and |*L*| denotes the number of locations; $d_{{\mathbf{f}}}$ denotes the maximal degree of the polynomials in the vector fields; *t* is the dimension of the matrix $\mathcal {B}({\mathbf{u}},{\mathbf{v}})$, and *N* refers to the number of decision variables appearing in the BMI problem (9), namely, $\dim ({\mathbf{u}})+\dim ({\mathbf{v}})$; $d_s$, $d_p$ and $d_l$ denote the degrees of the barrier certificates obtained via SISBMI, PENBMI and SOSTOOLS, respectively; $I_s$ and $I_p$ are the numbers of iterations used by SISBMI and PENBMI, respectively; $T_s$, $T_p$ and $T_l$ record the time spent by computation in seconds; the symbol—means that the solver was unable to return a feasible solution with the degree bound $\deg (B)\le 6$.

Table 1 shows that for the 19 examples, our SISBMI solver can successfully handle 17 of them while the numbers of successful examples of PENBMI and SOSTOOLS are 13 and 9, respectively. Our SISBMI solver seems to provide the best solving capability. There are 10 examples that can be treated by BMI solvers (either SISBMI or PENBMI) unable to be solved by the LMI solver SOSTOOLS due to the more conservative conditions in the corresponding LMI problems. To evaluate the best performance of SOSTOOLS, we have tried some widely used multipliers [16, 20], such as $0,\pm 1,\pm (1+x_1^2+\cdots +x_n^2)$, as well as some polynomial multipliers with random coefficients and the prior degree bound that guarantee the degrees of the polynomials involved in the verification conditions (6) do not increase. Examples C8-C9 show the case where the solver PENBMI performs better than our SISBMI solver as a result of the fact that both SISBMI and PENBMI solvers only find local optimal solutions to the BMI problems.

The above analysis on effectiveness can also be used to support that our SISBMI solver is a necessary complement to the existing tools. As shown in Table 1, PENBMI solver can cover 13 examples. To solve the remaining 6 examples, it has to resort to the SISBMI solver.

Considering the efficiency, the solver SOSTOOLS performs the best for almost all the successful examples because of the much lower computational complexity for solving the relaxed LMI problems. The efficiency comparison between SISBMI and PENBMI solvers can be made by examining the ratio between the execution times of these two solvers in Table 1. For the 11 examples that are solved by both tools, on average, our SISBMI solver costs 3.4 times than PENBMI solver in the number of iterations while only costs 0.27 times than PENBMI solver in time. That is for all the successful examples, our SISBMI solver takes much less time than PENBMI solver even it spends more iterations, which complies with the complexity analysis of the underlying algorithms. Both the theoretical analysis and the experiments support that our SISBMI solver is more efficient than PENBMI solver.

## Related Work

In theory, the problem of barrier certificate generation is a quantifier elimination problem. The verification conditions corresponding to a barrier certificate can be encoded into a set of constraints on state variables and coefficients where the unknown coefficients are existentially quantified and state variables are universally quantified. Hence, several symbolic computation approaches [11, 19, 29], such as cylindrical algebraic decomposition (CAD) or Grönber bases computation, have been directly applied to attack the associated quantifier elimination problems. However, due to the high computational complexity, they suffer from the scalability problem.

Due to the relatively low computational complexity, SOS relaxation based methods become popular. Rather than directly handling quantified constraints, they transform them to a non-convex bilinear matrix inequality. Z. Yang et al. [35] relied on the BMI solver PENBMI to compute exact polynomial barrier certificates. O. Bouissou et al. [3] applied interval analysis to handle the BMI problem derived from the dynamical systems whose initial and unsafe regions are restricted to the box form. G. Jessica et al. [10] presented an augmented Lagrangian framework for the special case of bilinear programs that arise from data flow constraints and correspond to the construction of numerical abstract domains aiming at safety verification.

To alleviate its computational intractability, a convex surrogate has been proposed that behaves fairly well. Specifically, once the multipliers are fixed, the BMI problem is further transformed into a LMI problem that can be quickly solved by convex optimization. S. Prajna et al. [20] had first put the idea forward. A. Sogokon et al. [34] employed the comparison principle associated with the convex verification conditions, to generate vector barrier certificates in safety verification.

Inspired by the fact that it is the non-convex feature of verification conditions prevents well-developed convex optimization to be directly applied, many convex but stronger verification conditions are studied. H. Kong et al. [16] proposed an exponential condition for semi-algebraic hybrid systems. Kapinski et al. [12] diagnosed convex verification conditions to Lyapunov-based barrier certificates. C. Sloth et al. [32] considered convex barrier certificates associated with compositional conditions for a group of interconnected hybrid systems. L. Dai et al. [4] studied how to balance the convexity of verification conditions with the expressiveness of barrier certificates. All these convex verification conditions are equivalent forms of LMI problems. They facilitate problem-solving at the risk of losing feasible solutions.

## Conclusion

We have presented a sequential iterative scheme for solving the BMI problem derived from the barrier certificate generation of semi-algebraic hybrid systems. Taking advantage of the special feature of the bilinear terms, the proposed approach is more efficient than the existing BMI solver. Furthermore, compared with popular LMI solving based methods, the solving procedure does not make the verification condition more conservative, and thus reduces the risk of missing solutions. In virtue of the two appealing features, our approach can produce barrier certificates not amenable to existing methods, which is evidenced by a theoretical complexity analysis as well as the experiment on some benchmarks.
